# Acute Kidney injury of glomerular origin in Asian population: Causes and outcomes - Meta-Analysis

**DOI:** 10.12669/pjms.41.5.11858

**Published:** 2025-05

**Authors:** Sakina Abbas, Aleena Aftab, Ahya Aziz, Rubina Naqvi

**Affiliations:** 1 Sakina Abbas, MBBS Sindh Institute of Urology and Transplantation (SIUT), Karachi - 74200, Pakistan; 2 Aleena Aftab, MBBS Sindh Institute of Urology and Transplantation (SIUT), Karachi - 74200, Pakistan; 3 Ahya Aziz, MBBS Sindh Institute of Urology and Transplantation (SIUT), Karachi - 74200, Pakistan; 4 Rubina Naqvi, MBBS, MD, FISN Sindh Institute of Urology and Transplantation (SIUT), Karachi - 74200, Pakistan

**Keywords:** Acute kidney injury, Asia, Glomerular diseases, Meta-analysis, Outcome

## Abstract

**Objective:**

To collect all studies on acute kidney injury of glomerular origin, published from Asia, even with their limitations, and look for causes and outcome in this particular population.

**Method:**

As of June 2024, a comprehensive literature search was conducted in the databases of PubMed, Cochrane Library, Web of Science, Embase, Google Scholar and PubMed Central, for full-text articles in English language, describing original articles on acute kidney injury of glomerular origin published from Asia. There was no time limit set for searching year of publication. Combinations of key words used were ‘acute kidney injury’ or ‘acute renal failure’ or ‘acute kidney injury of glomerular origin’ or ‘glomerular diseases causing acute kidney injury’ along with using ‘Asia’. Data was extracted and analyzed.

**Results:**

Of the 21 studies subjected to detailed analysis, 10(47.61%) had been published from China, 4 (19 %) from India, 2(9.52%) from Hong Kong and 1 (4.76 %) each from Iran, Korea, Pakistan, Saudi Arabia and Sri Lanka. Overall, there were 4,077 patients with acute kidney injury of glomerular origin. There were 7(33 %) studies addressing isolated lupus nephritis, two had isolated AAV, 1 Anti-GBM disease, two acute GN, two Crescentic GN, two PIGN, while 5 (24%) studies included different varieties including lupus and other categories in their population. Majority of studies were retrospective cohorts except one prospective. Pattern of defining and classifying acute kidney injury varied in the studies. Need for renal replacement varied widely from 7-80 %. Complete recovery varied from 9 to 96 % and mortality 3-38 % in the analyzed studies.

**Conclusions:**

The number of acute kidney injury patients was considerable. Despite variations in definitions, study designs and outcomes, the meta-analysis provides useful information about the pattern of the major causes of glomerular diseases causing acute kidney injury in Asia.

## INTRODUCTION

Acute kidney injury (AKI), formerly called acute renal failure (ARF), is a sudden decline in kidney function that often can be reversed. It leads to waste accumulation, electrolyte imbalances, and reduced urine output, marked by a rapid rise in serum creatinine or decreased urine output. The severity can vary from mild dysfunction to severe failure, sometimes requiring renal replacement therapy (RRT), like dialysis. Identifying the cause of AKI is essential for effective treatment and improving patient outcomes. AKI can result from various causes, broadly categorized into pre-renal, intrinsic (renal), and post-renal factors.^1^

Glomerulonephritis has frequently been reported as cause of AKI from some parts of world,^2^ whereas from Asian countries particularly from Pakistan it has been reported to contribute 4% of total community acquired AKI.^3^ Glomerular disorders which can cause AKI may arise from immune-mediated disorders, infections, or systemic diseases like diabetes and lupus. The high prevalence of infections such as hepatitis B and C, which are associated with glomerulonephritis has been reported from Korea.^4^ Additionally, autoimmune diseases like systemic lupus erythematosus (SLE), more common in certain Asian populations, contribute to the increased risk of glomerular disease-induced AKI.^5^ AKI caused by glomerular diseases often leads to variable outcomes, heavily influenced by the severity of the underlying condition and the timeliness of treatment.

Studies indicate that from very low to moderately high i.e. 7-76% of patients with glomerular disease-induced AKI require RRT, during the acute phase due to significant loss of kidney function.^6^,^7^ Despite treatment, the recovery rates can vary widely. Complete renal recovery has been reported from 9-96% 8,9 4-75% of these patients may recover sufficient kidney function to discontinue dialysis within weeks to months.^9^,^10^ However, a substantial proportion, particularly those with severe or inadequately managed glomerular diseases, may experience end stage kidney failure, reported 4-44% in different studies.^11^,^12^ Considering the importance of timely referring and managing the patients with any sort of glomerulonephritis leading to AKI, we aimed to collect published studies on subject from Asia and perform a meta-analysis. So that awareness lacking about the seriousness of issue can be highlighted.

## METHODS

This Meta-Analysis was performed in accordance with Preferred Reporting Items for Systematic Reviews and Meta-Analyses (PRISMA) 2020 expanded checklist.^13^ During month of June 2024 search was made using following combinations on search engines PubMed/ PubMed Central, Cochrane, Elsevier, Science Direct, Embase, Web of Science and Google Scholar. {(“acute kidney injury” OR “AKI”) AND (“RPGN” OR “Rapidly progressive glomerulonephritis”) AND (“lupus nephritis”) AND (“ANCA positive vasculitis”) AND (“pauci immune glomerulonephritis”) AND (“Anti GBM disease”) AND (“Asian” OR “asian population”) OR “Afghanistan” OR “Bangladesh” OR “China” OR “India” OR “Pakistan” Or “Nepal” OR “Sri Lanka”)} AAz, SA and AAb independently completed the search. The relevant studies were imported to Endnote X9 (Clarivate Analytics, US). After removing duplicates, titles and abstracts were screened by AAz, SA and AAb for relevance. Full text of potentially relevant articles were then reviewed for eligibility.

### Inclusion Criteria:

- ***Was based on the following:*** language, study design, intervention, patient population, comparison, definition, and outcomes of interest.
- ***Language:*** English publications.
- ***Study design:*** eligible completed randomized clinical trials or observational studies.
- ***Patient population:*** Adults and Children with acute kidney injury of glomerular origin in Asia only.
- ***Definitions:*** AKI definitions used were RIFLE, KDIGO, abrupt decline in GFR or histological based.
- ***Intervention:*** need for renal replacement therapy.
- ***Causes:*** all types of glomerulonephritis (GN), whether labeled as acute GN, Crescentic GN, Post Infection GN (PIGN), ANCA associated vasculitis (AAV), anti GBM disease.
- ***Outcomes of interest:*** complete renal recovery, Chronic Kidney Disease (CKD), End Stage Renal Failure (ESRF), or mortality during acute illness.

### Exclusion Criteria:

- No clear definition of population and types of glomerular diseases.
- Duplicates of previous publications.
- Insufficient data for estimating mean difference (MD) with a 95% confidence interval (CI).
- Children and adults with glomerular disease but no AKI.
- Systematic reviews, comments, reviews, single-arm studies, case-controlled studies.

The three investigators (AAz, SA and AAb) then independently extracted data which comprised authors names, publication year, country from study published, study design, age, gender, definition of AKI, type of glomerular disease, need for RRT, duration of follow-up and outcome in categories of complete renal recovery, progression to CKD or ESRF and mortality. R studio (version 4.16-2) was used for statistical analysis of studies, including drawing of Forest and Funnel plot. Quality of included study was checked and it was scored 8-9 in all.

## RESULTS

### Literature Search Results:

**Fig.1:**
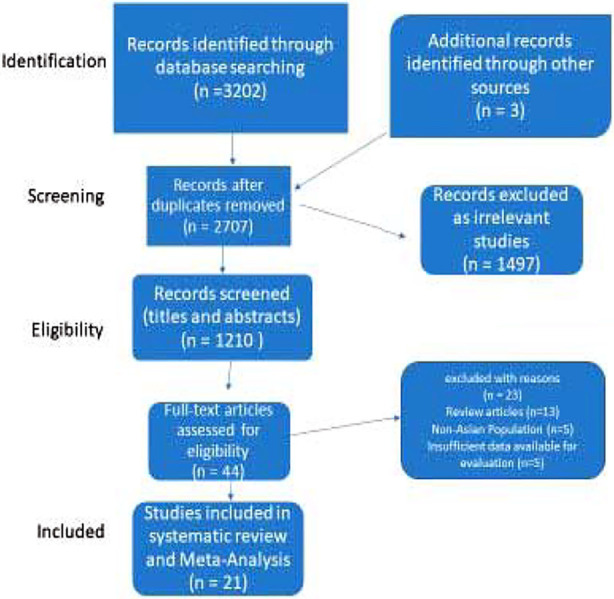
PRISMA Flow Chart.

Following an extensive search (done by three authors independently) across the following electronic databases: PubMed, PubMed Central, Embase, Google Scholar, Cochrane Library, Web of Science, a total of 3,205 records were identified. After the removal of 498 duplicate studies and 1,497 irrelevant records, 1,210 studies (abstracts) were assessed for eligibility. Subsequently, 44 studies found eligible, underwent full-text review and ultimately 21 studies were included in the systematic review and meta-analysis. The PRISMA flow diagram of the study selection procedure is summarized in Fig.1.

### Study Characteristics:

Among the 21 studies retained after screening and full-text review, ten originated from China, four from India, two from Hong-Kong, and one each from Iran, Pakistan, Korea, Saudi Arabia and Sri Lanka. The studies included were published between 1989 and 2024. The studies varied in sample size and event rates contributing to a comprehensive analysis of a total of 4,077 participants with a female: male ratio of 1.32:1 (Females=57.06%, Males=42.93%). In the selection of 21 studies, only one was a prospective observational study, while the remaining 20 studies were retrospective observational studies. The average age of the subjects differed across all studies, primarily encompassing young adults to middle-aged participants. The primary characteristics of the articles are shown in Table-I.

**Table-I:** Characteristics of included studies.

Author	Year of Publication	Study design	Study population	Country	Gender n(%)	Age in years mean±sd (range)	Glomerular injury	Duration of follow-up (months)	Definition of AKI	Need for RRT (%)	Outcome
Alexander S^12^	2020	observational retrospective cohort	200	India	M=80 (40) F=120(60)	40.6±14.6	Mixed (Lupus=41AAV=38 ANCA neg vas =37 PIGN=23 IgA=21 Anti GBM=21Dual Anti GBM and AAV=10 MPGN=4C1q Neph=3 HSP=1)	9.4±15	Histological presence of crescents	87 (43.5)	Rec=43 (21.5) CKD=60 (30) ESRF=88 (44) Died= 9 (4.5)
Chan PCK^8^	1989	observational retrospective cohort	11	Hong-Kong	M=0 F=11(100)	33.5	Lupus Nephritis	12-144 (mean 76.5)	Histology based	8(72)	Rec=1 (9) CKD=2(18) ESRF=4(36) Died=4 (36)
Chen S^16^	2016	observational retrospective	307	China	M=120 (39) F=187 (61)	37.6±16.4	Crescentic GN	120	Histological presence of crescents	216 (70)	NA
Chen XY^17^	2011	observational retrospective cohort	92	China	M=48 (52) F=44 (48)	57.7±16.75	AAV	12- 132	RIFLE Criteria	18 (20)	Rec=15 (16) CKD= NA ESRF=18 (20) Died=34 (37)
Chen Z^10^	2022	observational retrospective cohort	56	China	M=26 (46) F=30 (54)	54.29±15.90	AGN	12-96	Histological presence of crescents	40 (71)	Rec=NA CKD=42 (75) ESRF=NA Died=5 (9)
Fatemi A^18^	2013	observational retrospective cohort	82	Iran	M=17 (21) F=65 (79)	32.3± 11.4 (13–69)	Lupus nephritis	96	Creatinine >1.5 mg/dl	NA	Rec=55 (67) CKD=18(22) ESRF=4(5) Died=5 (6)
Gandhi TS^19^	2024	observational prospective	100	India	M=56 (56) F=44 (44)	40-60	PIGN	7.8	Abrupt reduction in GFR	23 (23)	Rec=54 (54) CKD=34 (34) ESRF=9 (9) Died=3 (3)
Ge H^9^	2020	observational retrospective cohort	26	China	M=18 (70) F=8 (30)	13 (10-15)	AGN	16-73	Abrupt reduction in GFR	0	Rec=25 (96) CKD=1 (4) ESRF=0 Died=0
Hari P^6^	2009	observational retrospective cohort	54	India	M=14 (26) F=40 (74)	10.4 ± 2.6 (3-15)	Lupus Nephritis	6-123	Abrupt reduction in GFR	4 (7.4)	Rec=35 (65) CKD=6 (11) ESRF=4 (7.4) Died=9 (16.6)
Herath N^20^	2017	observational retrospective cohort	20	Sri Lanka	NA	28 (13–60)	Lupus nephritis	12-72	Doubling of serum creatinine	NA	Rec=NA CKD=2 (10) ESRF=1 (5) Died=0
Huang X^21^	2021	observational retrospective	141	China	M=65 (46) F=76 (54)	59.6 (median)	AAV	64	KDIGO	56 (40)	Rec=19(13.4) CKD=NA ESRF=36 (25.5) Died=22 (15.6)
Jin SY^22^	2017	observational retrospective cohort	242	China	M=55 (23) F=187 (77)	11.7 ±2.8	Lupus Nephritis	21.5 ±18.4	Abrupt reduction in GFR	35 (14.46)	Rec=123 (71) CKD=16 (9) ESRF=25 (14.5) Died=7 (3)
Koubar SH^5^	2019	observational retrospective	55	China	M=13 (24) F= 42 (76)	29±11	Lupus Nephritis	120	Rise of creatinine of 0.3 mg/dl from baseline	8 (15)	Rec=11 (20) CKD=15 (27) ESRF=29 (53) Died=0
Lee H^15^	2013	observational retrospective cohort	1943	Korea	M=911 (47) F=1032 (53)	median 42	Mixed	median 90	Abrupt reduction in GFR	325 (16.7)	Rec=NA CKD=NA ESRF=325 (16.7) Died=164 (8.4)
Li FK^23^	2004	observational retrospective cohort	10	Hong Kong	M=2 (20) F=8 (80)	58.6 ± 21.7	Anti-GBM disease	12 -144	Abrupt reduction in GFR	8 (80)	Rec=1 (10) CKD=1 (10) ESRF=6(60) Died=2(20)
Mosaad FG^24^	2018	observational retrospective cohort	19	Saudi Arabia	M= 13 (68.5) F=6 (31.5)	8.52±3.15	PIGN (63.2%), LN (21.1%)	12-120	pRIFLE	4 (21)	Rec=5 (26) CKD=10(53) ESRF=4 (21) Died=NA
Naqvi R^7^	2015	observational retrospective	236	Pakistan	M= 103 (43.6) F= 133 (56.4)	27.94 ± 12.79	Mixed (GN with Crescents=118 GN without Crescents=78 AAV = 19 Anti-GBM = 8 Lupus=20 PIGN=34 MCGN=8)	300	RIFLE Criteria	179 (76)	Rec=103 (44) CKD=46 (20) ESRF=NA Died=27 (11)
Shankar M^25^	2022	observational retrospective	114	India	NA	37.05 ± 18.26	PIGN	36	Histology based	18 (15.7)	Rec=78 (68.4) CKD=18 (15.7) ESRF=NA Died=NA
Tang Z^11^	2009	observational retrospective cohort	94	China	M=10 (10.6) F= 84 (89.4)	27.9 ± 10.7	Lupus nephritis, Crescentic GN	37.9±38.5	Histological presence of crescents	NA	Rec=NA CKD=NA ESRF=4 (4.2) Died=5 (5.3)
Wang Y^26^	2005	observational retrospective	209	China	M=124 (59) F= 85 (41)	56.4±16.1 (18-92)	Crescentic GN in 36%of Biopsies	120	Rise of creatinine upto 2 mg/dl over hours or days	NA	Rec=97 (46) CKD=NA ESRF=32 (15) Died=80 (38)
Zhu D^27^	2011	observational retrospective cohort	66	China	M= 18 (27) F= 48 (73)	33.3±11.9	Lupus nephritis	53.6±57.1	Abrupt reduction in GFR	NA	Rec=28 (51) CKD=11 (20) ESRF=15 (27) Died=1 (2)

### Analysis of the Outcomes of AKI Secondary to Glomerular causes (Fig.2):

**Fig.2:**
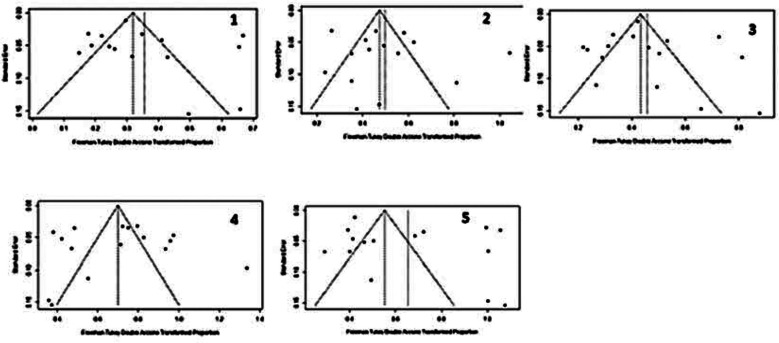
Forest plots for all-cause mortality. (l), CKD (2), ESRF (3), Complete Renal Recovery (4) and need for Renal Replacement Therapy (5). The red squares indicate point estimate of the effect of individual study with size representing weight of study and the horizontal lines represent the 95% confidence interval (CI). The dotted line represents the pooled estimate, and the diamond represents the overall effect estimate of meta-analysis. Heterogeneity outlined at near the bottom of forest plot.

***i. ESRF:*** In a collective analysis of 16 studies encompassing 3,338 patients, the likelihood of end-stage renal failure (ESRF) following acute kidney injury (AKI) attributable to glomerular causes was evaluated. The risk of ESRF development was assessed using both fixed-effect and random-effects models, yielding odds ratios (OR) of 0.17 (95% CI= 0.15;0.18) and 0.19 (95% CI = 0.12;0.27) respectively. These findings suggest that individuals experiencing AKI stemming from glomerular injury are at a substantial risk of progressing to ESRF.

***ii. CKD:*** A total of 15 studies involving 1,291 patients assessed the risk of progression to CKD after AKI of glomerular origin. The risk of CKD progression assessed using both fixed-effect and random-effects models was 0.20 (95% CI= 0.18;0.22) and 0.22 (95% CI= 0.14;0.32), respectively, suggesting 18% risk of CKD progression in patients with glomerular injury leading to AKI.

***iii. Complete Renal Recovery:*** A total of 16 studies involving 1,657 patients assessed the likelihood of complete renal recovery after glomerular injury has caused AKI, the OR (95% CI) under fixed-effect and random-effects models was 0.41 (0.39; 0.43) and 0.41 (0.28; 0.54), respectively, indicating 29% odds of Complete Renal Recovery after glomerular injury induced AKI.

***iv. Renal Replacement Therapy:*** A total of 15 studies involving 3,580 patients assessed the probability of Renal Replacement Therapy (RRT) requirement, revealing the OR (95% CI) of 0.27(0.25; 0.28) and 0.36 (0.23; 0.51) under fixed-effect and random-effects models respectively.

***v. All-Cause Mortality:*** A total of 15 studies involving 3,536 patients assessed for the extent of All-Cause Mortality after AKI originated by Glomerular injury, the OR (95% CI) under both fixed-effect and random-effects models was 0.09 (0.08; 0.10) and 0.11 (0.06; 0.18), respectively.

### Analysis of the Funnel Plots (Fig.3):

A funnel plot was constructed for each outcome by plotting the effect estimates from individual studies against their standard errors. The symmetry of the plot was assessed visually. All the funnel plots demonstrate considerable asymmetry, indicating significant heterogeneity and variability in the effect sizes. The asymmetry suggests potential biases, which may include publication bias or small-study effects.

**Fig.3:**
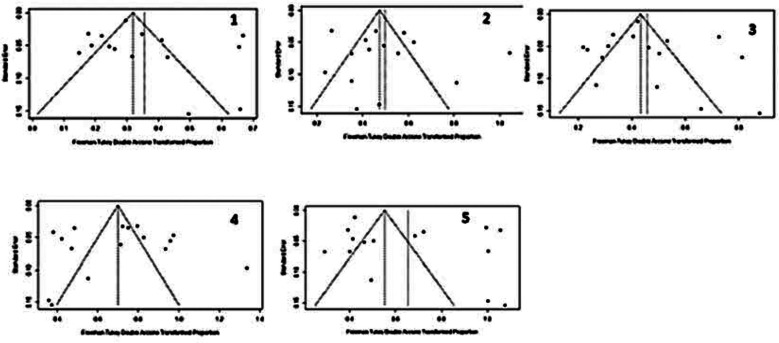
Funnel Plots for different outcomes and need for RRT. 1= Mortality, 2= CKD, 3= ESRF, 4= Complete Renal recovery, 5= Need for RRT.

## DISCUSSION

It is difficult to estimate actual burden and different causes of AKI, and outcome for these patients in Asian countries as many of these are lying in category of low income or lower middle income; according to World Bank classification based on per capita income, updated in July 2022.^14^ Budget spent on health care is very low in these countries and in majority of countries there is no proper state based health care provision. This results many of patients not reaching to proper nephrology set-up, those who reach tend to disappear amidst treatment mainly because of financial reasons. Furthermore, treated patients records are not maintained and health care providers lack facility of keeping records and enough funds to support publishing the results. This left us with very few published studies on topic of interest and hence lack of knowledge about complexity of disease and validity of management protocols.

Glomerular diseases causing AKI, in comparison to other cause of tubular or interstitial origin, have been reported to reveal high frequency of leading to CKD and over time eventually ESRF.^12^,^15^ This cause further burden on health budget of individual as well as state. For present meta-analysis extensive literature search on multiple search engines and giving the time and age limit, after removing duplicates and extracting through eligibility criteria we only left with 21 original studies on topic of interest. Even among these 21 some were lacking bit information about details of outcome.

Current study first of its kind that is addressing in a meta-analysis the AKI of glomerular origin from Asian continent. This meta-analysis highlights the following points; firstly, with heterogeneity of 91-95% among outcomes we found that wide variety among stages of AKI has been reported in different studies included in current meta-analysis. So is evident for management as supported with heterogeneity of 98% for need of RRT. Secondly, up to 44% patients registering as AKI at base line developing ESRF^12^, this is of serious note for nephrologist as well as health care community as a whole and affects economic states in health care. As patients with ESRF will be requiring lifelong RRT in one or other form.

In many of Asian countries RRT is not supported by insurance program or by state thus contribute in invisible rise in number of deaths under nephrology care patients. Funnel plots in current analysis were meant more for generic means of examining small study effects, rather as a tool to detect specific types of bias. As every center implies own definition of AKI and people do not tend to report outcome on any universally described outline, thus biases among studies is expected in such kind of analysis. The studies included in present meta-analysis revealed variations in definitions of AKI,^5^-^12^,^15^-^27^ some considered abrupt rise in creatinine, some as doubling of serum creatinine from baseline, others mentioned serum creatinine of more than 2 mg/dl, some described abrupt fall in GFR, some considered histological pattern of acute cellular crescent formation with few using RIFLE or KDIGO guidelines. Duration of follow up has also revealed a variation from shortest of 32 weeks to longest 25 years (Table-I).

Variety of glomerular lesion seen in this meta-analysis. Some of studies addressing only one aspect of glomerular disease that is lupus nephritis,^5^,^6^,^8^,^18^,^20^,^22^,^27^ one study only addressed anti GBM disease,^23^ some addressing only acute glomerulonephritis,^9^,^10^ isolated AAV in two,^7^,^21^ Crescentic GN in two,^16^,^26^ PIGN in two^19^,^25^ and rest of studies had mixed causes for glomerular lesions including one or more one of above.^7^,^11^,^12^,^15^,^24^ The largest population study included in current meta-analysis^15^ was actually comprised of all biopsied cases over a median follow up of 90 months and addressed the outcome for those who developed ESRF or died during follow-up period but complete renal recovery and number of CKD is not mentioned in this large cohort.

Another large study from China reporting study population of 528 patients with biopsy proven crescentic glomerulonephritis (GN), has shown variation in causes of GN, including AAV and anti-GBM disease, 58% of their studied population developed AKI, while another 13% they categorized as acute kidney disease (AKD). In this study worse prognosis was observed in anti-GBM disease related AKI.^16^ Moreover, study published in past from our center has also shown worst prognosis towards renal recovery from same type of GN, that is, anti-GBM disease.^7^ Dual positivity of ANCA and anti-GBM at same time is reported as occurring at older age, causing more severe renal damage and resulting in high mortality.^7^,^16^ Another study published from China, with smaller study cohort, using same definitions of histologic pattern and crescentic GN, also had AAV and anti-GBM positive patients, this particular study has not mentioned number of renal recovery but mortality rate was 9% in their studied population.^10^

Only prospective study in this meta-analysis, comprising of 100 patients was addressing infection related GN, where most common source (41%) of infection was skin and soft tissues. Histological pattern in this study was 63% diffuse proliferative GN, 22% endo-capillary proliferative GN, 8% crescentic GN with >50% glomeruli with crescents and another 28% having crescents in <50% glomeruli. In their studied population 3% died during acute illness, 34% remained CKD and 9% dialysis dependent (ESRF), 26 % had persistent hypertension, the follow up was six months in this particular study.^19^ Heterogeneity among mortality highlights the differences in stages of AKI at time of presentation and possible impact of immunosuppression (effect of this factor not analyzed) used in these patients.

### Limitations:

Scarcity of studies published from region and discrepancies in way of defining and addressing the outcomes, as well as almost nonexistence of well-designed prospective cohorts were main limitations during this analysis. Heterogeneity with *I*^2^ of up to 98% is also a limitation towards setting down any hard core recommendation. However, heterogeneity was inevitable because these studies were reported from different centers of countries in Asia with varying type of set up of nephrology services and available facilities.

## CONCLUSIONS

In conclusion; our analysis included more than 4,000 patients from 21 studies, reflecting major causes of AKI related to glomerular diseases in the region. All studies were observational with wide heterogeneity. This study provides a wide spectrum of glomerular diseases causing AKI, highlighting important disparities related to availability of nephrology care in different Asian countries which include both lower income and lower middle income countries. Future work in this field, in this region, should focus on designing prospective or randomized multicenter studies targeting standard definitions of AKI and proper documentation of events during management as well as follow up.

### Authors contributions:

**SA:** Intern, Department of Nephrology, SIUT, did search independently through different search engines, entered and analyzed the results.

**AAB and AAZ:** Intern, Department of Nephrology, SIUT, did search independently through different search engines, entered the data, written introduction and results.

**RN:** Professor, Department of Nephrology, SIUT, given concept, guided through selection of articles, reviewed articles included in final analysis, written discussion and responsible for accuracy of study.

All authors have read and approved the final manuscript.
